# Stress Physiology and Molecular Biology of Fruit Crops

**DOI:** 10.3390/ijms25020706

**Published:** 2024-01-05

**Authors:** Lin-Tong Yang, Li-Song Chen

**Affiliations:** College of Resources and Environment, Fujian Agriculture and Forestry University, Fuzhou 350002, China

Fruit crops provide various kinds of fruit commodities that are of significant nutritional benefit and economic value to humans [1]. Fruits provide essential and valuable nutrition to humans, and they have an increasingly crucial role in helping humans respond to numerous disorders and diseases [2]. The beautiful colors and fragrant scents of different fruits well fulfil our aesthetic and sensory enjoyment. Currently, fruit commodities with good appearance and high quality bring us considerable economic interest. However, fruit quantity, appearance, and nutritional qualities, including the contents of their diverse primary and secondary metabolites, namely amino acids, organic acids, flavonoids, anthocyanins, soluble sugars, vitamins, etc., are determined by the growth status of fruit trees, including the phenotypes dictated by genetic characteristics, tree nutrition, and biotic and abiotic stimuli [2,3]. So far, numerous pieces of literature, including those in this Special Issue, have highlighted the physiological and molecular response of the abiotic stresses, new progresses of physiological response, and underlying molecular mechanisms of fruit crops in response to environmental stresses, such as mineral nutrient deficiencies and toxicities, osmotic stress, heavy metal stress, temperature stress, and light stress, as well as the strategies aiming at improving the stress tolerance of fruit crops and selecting the resistant varieties by screening the biomarker compounds and resistance molecular breeding [4,5,6,7,8,9].

Under the condition of global warming, with more frequent occurrences of extreme weather events, including exceptionally hot weather, drought, and/or rainstorms, the adverse effects of high temperatures, drought, and/or waterlogging on fruit growth and agricultural production are becoming increasingly severe, and they compromise both fruit yield and fruit quality [10]. Detrimental weather conditions can cause reductions in fruit crop growth, affecting yield quality and quantity, and they can result in significant fruit losses. High-temperature-induced morphological, physiological, and molecular disorders have been reported in fruit trees [3]. High temperatures can affect fruit tree growth and fruit characteristics in various aspects, including the flowering number, fruit drop and fruit deformities, and the slowdown of fruit growth. Drought also affects the normal growth of fruit crops. Zhang et al. showed that altered expression levels of genes related to photosynthesis, carbohydrate metabolism, oxidoreductase activities, nutrient metabolism, and senescence pathways were observed in drought-treated seedlings. Furthermore, the contents of some plant hormones, such as gibberellin and abscisic acid, were affected in drought-treated seedlings. This suggested that a widespread impact of drought existed in fruit crops [10]. Waterlogging is another constraining factor for fruit crops. Zhang et al. reported that differentially expressed genes (DEGs) were differentially regulated between two apple cultivars (waterlogging-tolerant *Malus hupehensis* and sensitive *M. toringoides*) under waterlogging stress, especially those DEGs involved in flavonoids metabolism and hormone signaling, suggesting a possible link between flavonoids and hormone signaling and waterlogging tolerance [11]. An experiment using kiwifruit plants under waterlogging and drought demonstrated that the contents of abscisic acid (ABA) and ABA responsive or biosynthesis genes, such as DRE-binding protein 2 (*DREB2*), “W-tryptophan, R-arginine, K-lysine, Y-tyrosine 40” (*WRKY40*), nine-cis-epoxycarotenoid dioxygenase 3 (*NCED3*), etc., were upregulated by these two water stresses, supporting the theory that kiwifruit plants can combat water stress extremes by regulating ABA metabolism and signaling [12].

Except for weather conditions, geochemical factors and agronomic management can also bring on unfavorable factors in the cultivation of fruit crops. For instance, Lu et al. showed that the application of the copper-containing pesticide Bordeaux mixture increased the contents of leaf copper (Cu), decreased photosynthetic pigments, and decreased the efficiency of photo-electron transport, inducing leaf chlorosis and photosynthetic inhibition. The Cu-induced impairment of chloroplast ultrastructure and enhancement of antioxidant systems were different between *Citrus grandis* and *C. sinensis*, which conferred a higher tolerance of Cu toxicity to *C. grandis* [9]. Some new biomarkers involving nutrient stress have been identified in the literature. For example, Liu et al. found that the overexpression of SlmiR319b-regulated *Teosinte-Branched1*/*Cycloidea*/*PCF* 10 (*SlTCP10*), which mediates *Jasmonic Acid 2* (*SlJA2*) in roots, can enlarge root growth and potassium (K) absorption in tomato [13]. The overexpression of *Malus domestica Auxin*/*Indole-3-Acetic Acid 27* (*MdIAA27T*) can improve the tolerance to phosphorus (P) deficiency in transgenic apple trees by growing longer and denser adventitious roots and taking up higher P content than the control plants under low-P conditions [14]. These studies could provide a new regulation mechanism for increasing K and P acquisition efficiency under low-K and low-P stress.

Soil acidification caused by strong base leaching and desilicification/allitization in tropic and subtropical areas consequentially led to several metal stresses, such as aluminum (Al) and manganese (Mn) stress and nutrient deficiency. Huge amounts of work have put forward extensive details of Al toxicity in fruit crops cultivated in acidic soil [15,16]. Physiological, transcriptomic, and proteomic studies have shown that both Al and Mn can affect the integrity of the cell wall and plasma membrane and disrupt the normal cellular processes of nucleic acids, amino acids, carbohydrates, energy metabolisms, growth regulation, and signal transduction [15,16,17,18]. To cope with Al toxicity, some plant species and cultivars have evolved mechanisms to detoxify Al, both externally and internally, through the exudation of organic acids or other secondary metabolites and the chelation of Al cations into nontoxic forms [17]. In *citrus* plants, the enhancement of reactive oxygen species (ROS) metabolism and sulfur compound metabolism has also been proven to play important roles in Al tolerance [19]. To effectively eliminate Al toxicity, except for liming, it is proposed that the application of some beneficial compounds such as melatonin, methyl jasmonate, and sulfur-containing fertilizer in the field could be practicable in the cultivation of fruit crops [20,21]. In fields, the inappropriate application of chemical fertilizer and neglecting the application of organic manure can also lead to nutrient disorders such as magnesium deficiency, potassium deficiency, zinc deficiency, and boron stress in orchards [8]. Meanwhile, considering the complexities of outdoor conditions, more than one unfavorable factor is simultaneously applied to fruit crops, increasing the difficulty of finding countermeasures. Hence, a better understanding of the response mechanisms of fruit crops to adverse conditions is hugely significant for the sustainability of fruit production.

In conclusion, the products of fruit crops are agricultural commodities of great biological and economic importance. Sustainable agriculture is, today, both a challenge and a necessity for increasing the demands of the continually growing population. Understanding responses and how to manipulate stresses will help increase fruit crops’ resilience and productivity during adverse conditions. Therefore, precise knowledge of physiological and molecular mechanisms for high-quality production and resistance molecular breeding that boost fruit production is of great importance.
